# Expression of HER2 in high-grade urothelial carcinoma based on Chinese expert consensus and the clinical effects of disitamab vedotin-tislelizumab combination therapy in the treatment of advanced patients

**DOI:** 10.3389/fphar.2024.1355081

**Published:** 2024-02-22

**Authors:** Kejia Zhu, Yao Chang, Delong Zhao, Andong Guo, Jishuang Cao, Chenrui Wu, Yong Guan, Sentai Ding

**Affiliations:** ^1^ Department of Urology, Shandong Provincial Hospital Affiliated to Shandong First Medical University, Jinan, Shandong, China; ^2^ Medical Integration and Practice Center, Cheeloo College of Medicine, Shandong University, Jinan, Shandong, China; ^3^ Department of Urology, Liaocheng People’s Hospital, Liaocheng, Shandong, China; ^4^ Engineering Laboratory of Urinary Organ and Functional Reconstruction of Shandong Province, Shandong Provincial Hospital Affiliated to Shandong First Medical University, Jinan, Shandong, China; ^5^ Department of Urology, Central Hospital Affiliated to Shandong First Medical University, Jinan, Shandong, China; ^6^ Department of Urology, Shandong Provincial Hospital, Cheeloo College of Medicine, Shandong University, Jinan, China

**Keywords:** urothelial carcinoma, high-grade, HER2, antibody-drug conjugate, clinical significance, prognosis, pathology

## Abstract

**Background:** A vast number of researchers have discovered high levels of human epidermal growth factor receptor-2 (HER2) expression in urothelial carcinoma (UC), but they do not use a uniform scoring system. Based on the 2021 edition of clinical pathological expert consensus on HER-2 testing in UC in China, we investigated the expression level and clinical significance of HER2 in high-grade UC. Furthermore, we looked at the prognosis of patients with locally advanced/metastatic UC after combining HER2 targeting antibody-drug conjugates (ADC) medication disitamab vedotin (DV) with programmed cell death protein 1 (PD-1) inhibitor tislelizumab.

**Patients and methods:** From 2019 to 2022, we collected paraffin specimens of UC from the Department of Urology at the Provincial Hospital Affiliated to Shandong First Medical University. HER2 expression-related factors were investigated. Patients with advanced UC who have failed systemic chemotherapy at least once and had received immune checkpoint inhibitor (ICI) medication during second-line treatment were selected and treated with DV in combination with tislelizumab. We assessed the therapy’s efficacy and safety.

**Results:** 185 patients with high-grade UC were included in this investigation. 127 patients (68.7%) were HER2 positive (IHC 2+/3+) according to the 2021 Clinical pathological expert consensus on HER2 testing in UC in China. The clinical stage of UC differed statistically significantly between the HER2-and HER2+ groups (*p* = 0.019). Sixteen advanced UC patients were treated with DV and tislelizumab for a median of 14 months. The disease control rate was 87.5%, while the objective response rate (ORR) was 62.5%. The ORR of HER2+ individuals was higher than that of HER2-individuals (70.0% vs. 50.0%). The median progression-free survival or overall survival was not reached. In this study, the incidence of treatment-related adverse events was 68.8% (11/16), with all of them being grade 1 or 2 adverse reactions.

**Conclusion:** HER2 protein expressed at a high percentage in UC, and 68.7% patients expressed HER2 positive (IHC 2+/3+). HER2+ expression is positively correlated with higher clinical stage of UC. HER2 targeted ADC drug disitamab vedotin combining with PD-1 inhibitor tislelizumab has shown efficacy, safety and controllable adverse reactions in the treatment of advanced UC.

## 1 Introduction

The urothelial carcinoma (UC) is one of the most prevalent cancers worldwide, with primary locations including the bladder, ureter, and renal pelvis. According to the Global Cancer Statistics in 2020, there were 573,278 new cases of bladder cancer globally and 85,649 in China (Sung et al., 2021). UC can be classified into low-grade UC and high-grade UC. High-grade UC is often associated with stromal invasion and has a poor prognosis. Locally advanced or metastatic (la/mUC) cases account for approximately 5%–11% of all UC cases (Hepp et al., 2021; He et al., 2023). Patients with la/mUC face a bleak prognosis, as the 5-year survival rate ranges from a mere 4.6%–34%. Therefore, there is an urgent need for an improved non-surgical treatment approach.

Currently, adjuvant therapy for advanced UC includes chemotherapy, immunotherapy, and targeted therapy. Chemotherapy is the recommended first-line treatment, while immunotherapy (particularly programmed cell death protein 1 (PD-1)/programmed cell death-ligand 1 (PD-L1) inhibitors) is a second-line option. Enfortumab vedotin antibody-drug conjugates (ADC) after prior platinum chemotherapy and checkpoint inhibitor immunotherapy (ICI) have demonstrated significant survival benefits in la/mUC patients compared to chemotherapy (Rosenberg et al., 2019; Chang et al., 2021). The efficacy and safety of the combination therapy comprising enfortumab vedotin and pembrolizumab as a first-line treatment in cisplatin-ineligible patients with la/mUC were also confirmed (Hoimes et al., 2023).

In recent years, an additional ADC targeting the human epidermal growth factor receptor 2 (HER2, also known as ERBB2) has been developed and implemented in clinical practice. It has been established that HER2 plays a crucial role in the pathogenesis and progression of various malignant tumors, including urothelial carcinoma. Consequently, it is imperative to elucidate the expression of HER2 protein in urothelial carcinoma and its clinicopathological correlation to facilitate the clinical application of anti-HER2 targeted therapy for this disease (Olt et al., 1990; Press et al., 1990; Mendelsohn and Baselga, 2003). Currently, the existing detection methods for HER2 expression primarily rely on breast cancer evaluation standards that lack a standardized scoring system; thus further validation is warranted. Notably, several HER2-targeted ADCs have been approved in recent years, such as trastuzumab emtansine and trastuzumab deruxtecan. Additionally, a novel HER2-targeted ADC named disitamab vedotin (DV) was granted approval by the National Medical Products Administration in January 2022. However, more comprehensive clinical data are required to evaluate the efficacy of this medication.

In this article, we detected the expression of HER2 based on a 2021 edition of Clinical pathological expert consensus on HER-2 testing in UC in China. Besides, we analyzed its clinicopathological relationship with high-grade UC and explored the efficacy of HER2 targeted ADC drug disitamab vedotin, also known as RC48. As well as the efficacy of DV and PD-1 inhibitor tislelizumab combination therapy.

## 2 Patients and methods

### 2.1 Clinical patients

All clinical cases of high-grade UC with pathological diagnosis from 2019 to 2022 were included in this study, conducted at the Department of Urology and Pathology, Shandong Provincial Hospital Affiliated to Shandong First Medical University. The pathological diagnosis was based on the 2016 edition of the World Health Organization’s diagnostic criteria for urological pathology and genetics. UC specimens were obtained through various surgical procedures including transurethral resection of bladder tumor, partial cystectomy, radical cystectomy, segmental ureterectomy, or total ureteropelvic resection.

For evaluating the efficacy of DV-tislelizumab combination therapy, patients treated between 2020 and 2022 were included in our analysis. The inclusion criteria consisted of: 1) age ≥18 years; histological or cytological confirmation of la/mUC with at least one measurable tumor lesion; 2) Eastern Cooperative Oncology Group (ECOG) performance status ≤1; 3) previous failure with systemic chemotherapy allowed; and 4) prior treatment with PD-1/PD-L1 inhibitors permitted. Exclusion criteria encompassed: 1) insufficient availability of critical clinical data; 2) inability to detect HER2 expression or lack of pathological sections; 3) presence of concurrent malignancies; and finally; 4) history of tyrosine kinase inhibitor therapy.

### 2.2 Experiment design

The collected high-grade UC specimens were fixed within 1 h after isolation. Prior to fixation, the specimen was incised at intervals of 0.5–1 cm, and gauze or filter paper was inserted between the tissues for optimal fixation. In cases where the tumor tissue was fragmented, packing fixation was employed. A solution of 10% formalin, with a volume ten times that of the specimen, was utilized for fixation, with biopsy specimens being fixed for a duration ranging from 6 to 24 h. For larger specimens, fixation extended from 12 to 48 h. Subsequently, the paraffin-embedded specimens were subjected to HER2 immunohistochemistry (IHC) analysis following the scoring criteria outlined in accordance with the Clinical Pathological Expert Consensus on HER2 Testing in UC in China (2021 edition): no staining or <10% of invasive cancer cells exhibiting incomplete and weakly stained membranes (scored as 0); ≥10% of invasive cancer cells displaying incomplete and weakly stained membranes (scored as 1+); ≥10% of invasive cancer cells showing weak-moderate full membrane staining or <10% of invasive cancer cells demonstrating strong staining of intact cell membrane (scored as 2+); ≥10% of invasive cancer cells exhibiting strong staining of intact cell membrane (scored as 3+) (HERExpert Committee on Urothelial Carcinoma of Chinese Society of Clinical Oncology, 2021). Additionally, patient demographics including gender and age along with clinical parameters such as maximum tumor size, smoking history, primary sites, histopathological diagnosis, clinical stage classification, muscle invasion status, regional lymph node metastasis presence as well as some laboratory results were also recorded.

Locally-advanced/metastatic UC patients meeting the inclusion criteria were enrolled in this study to receive combination therapy with DV-tislelizumab. The treatment regimen consisted of DV administered at a dose of 2.0 mg/kg every 2 weeks, in combination with tislelizumab, a PD-1 inhibitor, administered at a dose of 200 mg every 3 weeks. Treatment was continued until patients discontinued due to disease progression (PD), intolerable side effects (SE), death, or withdrawal of informed consent. The patients underwent baseline physical examinations. Efficacy assessments were conducted every 4 treatment cycles, following the 1.1 version of the Solid Tumor Response Evaluation Standard and the International Standard for Common Terminology of Adverse Events (RECIST) (Eisenhauer et al., 2009). Short-term evaluation included objective response rate (ORR) and disease control rate (DCR) determination. Median progression-free survival (PFS) and overall survival (OS) served as long-term evaluation endpoints. During treatment, patients were monitored biweekly for blood routine tests (RT), biochemical parameters, liver functions, thyroid functions, stool and urine RT. Side effects were evaluated according to Common Terminology Criteria for Adverse Events (CTCAE) 5.0. Grade 1 or 2 side effects were managed based on symptoms, while grade 3 or higher side effects led to discontinuation from the study protocol. Informed consent was obtained from all participants, and ethical approval was granted by our institution’s ethics committee.

### 2.3 Statistical analysis

Chi-square test was applied to analyze the characteristics of different primary sites, and relationship between HER2 and UC muscle invasion, clinical stage, regional lymph node metastasis, smoke history, primary sites and gender. ANOVA test was used to analyze the difference among three primary sites. *t*-test and Mann-Whitney test were used to analyze the differences between HER2^+/−^groups. Logistic analysis was used for the correlation between HER2 and some blood indicators analysis. *p* < 0.05 was set as statistically significant. All analyses were performed using SPSS version 27.0 software.

## 3 Results

### 3.1 Clinical patients baseline

**FIGURE 1:**
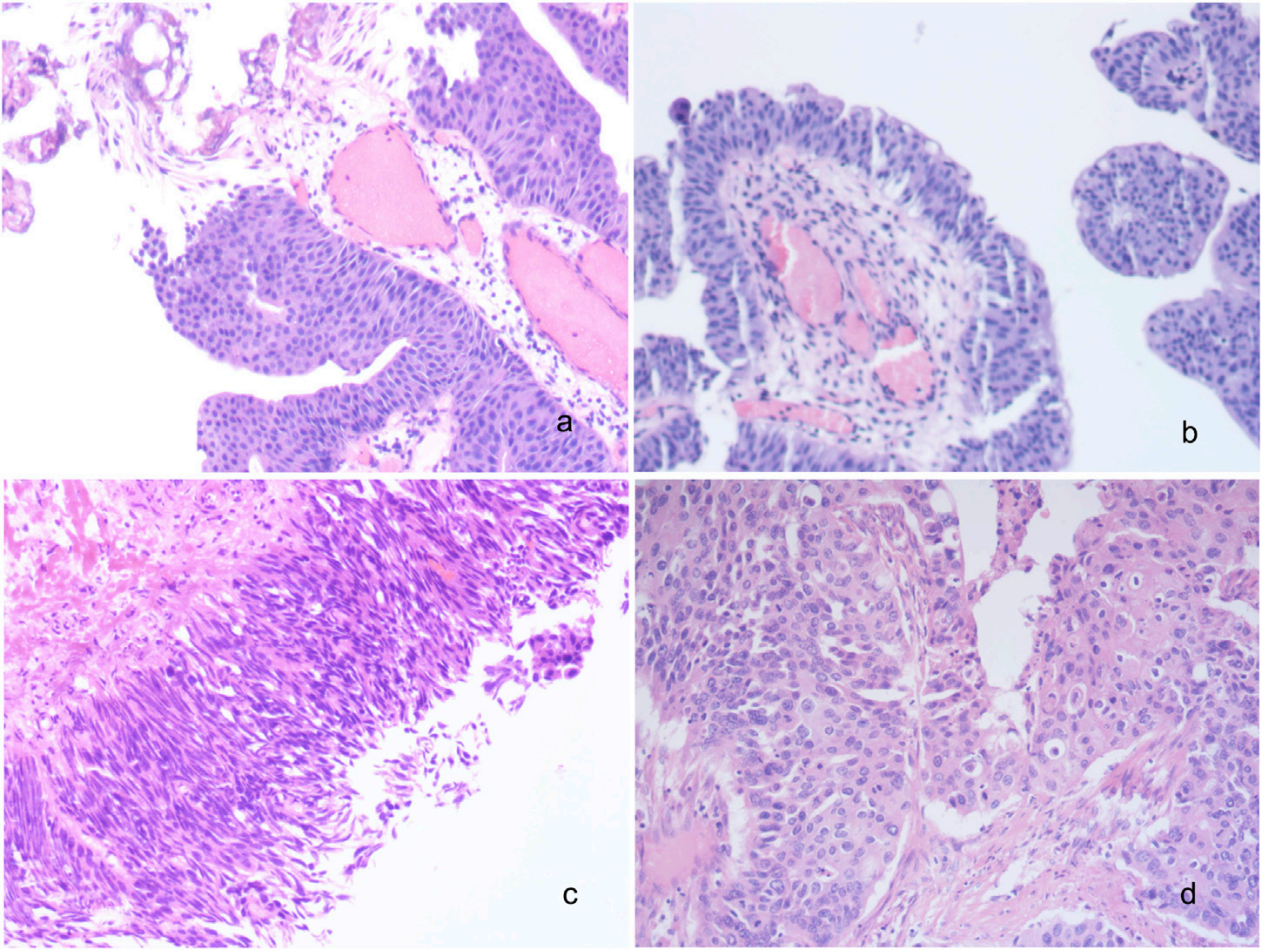
Histomorphological spectrum of UC. **(A)** Low grade non-invasive urothelial bladder carcinoma; **(B)** Low grade invasive urothelial bladder carcinoma; **(C)** High grade non-invasive urothelial bladder carcinoma; **(D)** High grade invasive urothelial bladder carcinoma; ×200. UC, urothelial carcinoma.

In our study, a total of 185 patients with high-grade UC were included, comprising 139 males and 46 females, with a median age of 68 years (range: 41–93). Among them, bladder was the primary site for UC in 127 cases (68.7%), followed by ureter in 36 cases (19.5%) and renal pelvis in 22 cases (11.9%) (Table 1). According to the diagnostic criteria outlined in the World Health Organization’s (WHO) latest edition from 2016, muscle invasive UC was observed in 103 cases (primary site: bladder-54; ureter-31; renal pelvis-18), while non-muscle invasive UC was found in 82 cases (primary site: bladder-73; ureter-5; renal pelvis-4) (Figure 1).

**TABLE 1:** Baseline clinicopathological features of the analyzed cohort [n, n (%)].

	Bladder	Ureteral	Renal pelvis	*P*
N	127	36	22	
Gender				
Male	113	20	6	<0.001
Female	14	16	16	
Age/years (Median)	61	70	68	0.386
Range	41–93	52–85	55–87	
Tumor size/cm				0.807
≥2	95	27	18	
<2	32	9	4	
Muscle invasion				<0.001
MIUC	54	31	18	
NMIUC	73	5	4	
pN				0.038
N1	8	4	5	
N0	119	32	17	
Stage				0.001
Ⅰ	72	14	4	
Ⅱ	22	10	3	
Ⅲ	24	6	8	
Ⅳ	9	6	7	

N, number; WHO, world health organization; pN, primary lymph node stage; MIBC, muscle invasive urothelial cancer; NMIBC, non-muscle invasive urothelial cancer.

Among the patients with primary tumor located in the bladder (*n* = 127), transurethral resection of bladder tumor was performed on 61 patients with a HER2 positive rate of 80.3%. Radical cystectomy was conducted on 50 patients with a HER2 positive rate of 46.0%, partial cystectomy on 14 patients with a HER2 positive rate of 64.3%, and no surgical treatment or bladder instillation but only immunotherapy using PD-1 inhibitor was administered to 2 patients who had a HER2 positive rate of 50.0%; For 36 patients with a primary tumor located in the ureter, radical nephroureterectomy was performed on 26 patients (HER2 positive rate: 50.0%), while segmental ureterectomy was performed on 10 patients (HER2 positive rate: 70.0%). Among the cohort of 22 patients with primary tumors located in the renal pelvis, a total of 19 patients underwent radical nephroureterectomy combined with partial cystectomy (with a HER2 positive rate of 57.9%). For the remaining 3 patients who did not undergo surgical intervention, PD-1 inhibitor immunotherapy was administered instead (with a HER2 positive rate of 33.3%).

### 3.2 Expression of HER2 in UC

**FIGURE 2:**
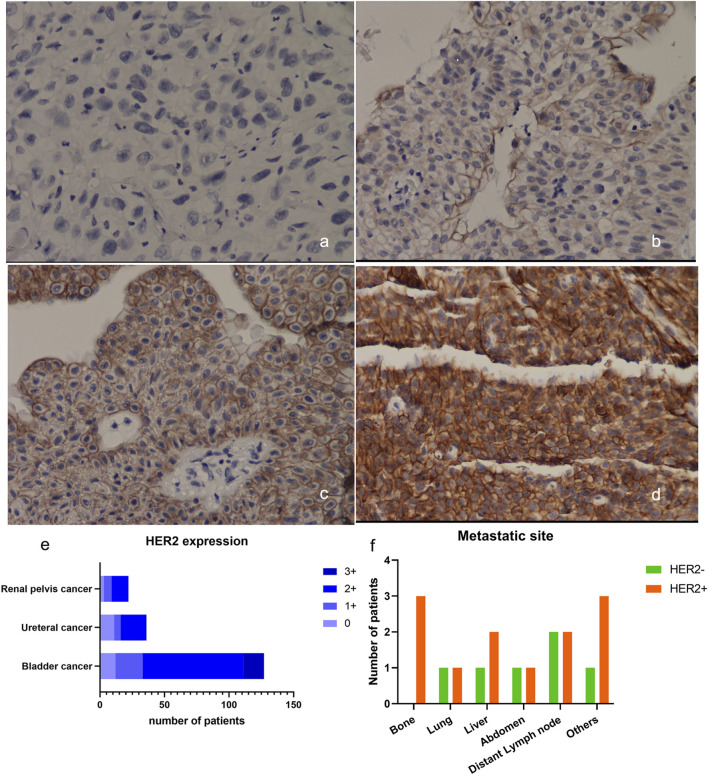
Expression of HER2 in UC. **(A–D)** Example of HER2 expression in urothelial bladder carcinoma. **(A)** HER2 IHC scored 0+; **(B)** HER2 IHC scored 1+; **(C)** HER2 IHC scored 2+; **(D)** HER2 IHC scored 3+, ×200; **(E)** Expression of HER2 in UC from different primary sites; **(F)** The metastatic sites of patients included in the DV treatment therapy at baseline. HER2, human epidermal growth factor receptor 2; IHC, immunohistochemistry.

Among the 185 high-grade UC tissues, HER2 protein expression was observed in 159 cases (86.0%) (Figures 2A–D). Of these, 32 cases (17.3%) showed HER2 1+ expression, while 110 cases (59.5%) exhibited HER2 2+ expression and only 17 cases (7.6%) displayed HER2 3+ expression. 127 of the analyzed samples were high-grade bladder tumor tissues, with a majority of them showing HER2 protein expression in 115 cases (90.6%), 21 (16.5%) expressed HER2 1+, 79 (62.2%) expressed HER2 2+ and 16 (12.6%) expressed HER2 3+; Additionally, 36 cases were high-grade ureteral tumor tissues, and among them 25 cases (69.4%) expressed HER2 protein. Of these, 5 cases (13.9%) had HER2 score of 1+, 19 cases (52.8%) had HER2 score of 2+ and only 1 case (2.8%) had HER2 3+. Among the 22 high-grade renal pelvis tumor tissues, HER2 protein was expressed in 19 cases (86.4%), with 6 cases (27.3%) showing HER2 1+ expression and 13 cases (59.1%) exhibiting HER2 2+ expression (Figure 2E).

### 3.3 Factors correlated with HER2+ in UC patients

**FIGURE 3:**
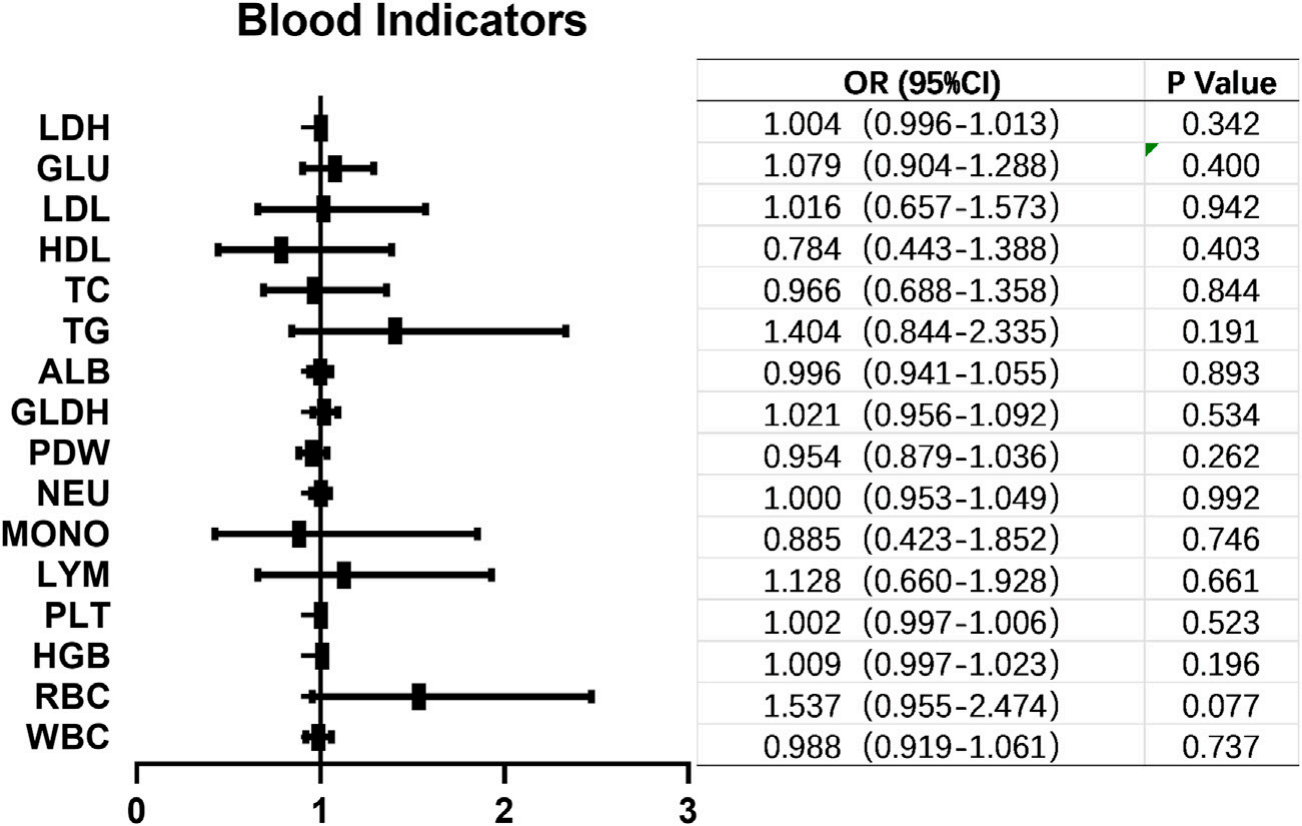
Blood indicators that might correlated with HER2+ in UC. No blood indicators showed correlation with HER2+ expression in UC. OR, odds ratio; CI, confidence interval; LDH, lactatedehydrogenase; GLU, glucose; LDL, low density lipoprotein; HDL, high density lipoprotein; TC, total cholesterol; TG, triglyceride; ALB, albumin; GLDH, glutamic dehydrogenase; PDW, platelet distribution width; NEU, neutrophil; MONO, monocyte; LYM, lymphocyte; PLT, platelet; HGB, hemoglobin; WBC, white blood cell.

The HER2 positive rate (IHC 2+ and 3+) in 185 high-grade UC patients was found to be 68.65% (127/185). We conducted an analysis to identify potential factors associated with HER2 positivity. Notably, clinical stage exhibited a statistically significant correlation with HER2 positivity in UC (*p* = 0.019). However, no significant associations were observed between HER2 positivity and gender (*p* = 0.345), age (*p* = 0.289), tumor size (*p* = 0.107), smoking status (*p* = 0.175), muscle invasion (*p* = 0.133), regional lymph node metastasis (*p* = 0.143) or primary site of the tumor (*p* = 0.066) as shown in Table 2. Furthermore, we investigated whether any blood indicators could predict the expression of HER2+. Logistic regression analysis revealed that none of the blood indicators examined showed predictive value for HER2+ expression (Figure 3).

**TABLE 2:** Relationship between HER2+ and some clinicopathological features of UC [n, n (%)].

	HER2+	HER2-	*P*
N(%)	127 (68.65%)	58 (31.35%)	
Gender			0.345
Male	98	41	
Female	29	17	
Age (years)	68 (41–93)	67 (43–85)	0.289
Tumor diameter (cm)	2.6 (0–10)	3.0 (0.8–8.5)	0.107
Smoke			0.175
Yes	50	29	
No	77	29	
Muscle invasive			0.133
Yes	66	37	
No	61	21	
pN			0.143
N1	9	8	
N0	118	50	
Stage			0.019
Ⅰ	64	26	
Ⅱ	24	11	
Ⅲ	30	8	
IV	91	13	
Primary site			0.066
Bladder	94	33	
Ureter	20	16	
Renal pelvis	13	9	

HER2, human epidermal growth factor receptor 2.

### 3.4 The efficacy of DV on the treatment of HER2+ UC patients

Sixteen patients with locally-advanced/metastatic UC, who had failed first-line treatment, were enrolled in this study. They received DV 240 mg every 2 weeks in combination with PD-1 inhibitors tislelizumab 200 mg every 3 weeks. The cohort consisted of an equal distribution of male and female patients, with a median age of 66 (range: 51–81) years old. Metastatic sites included the liver (*n* = 3), lung (*n* = 2), and bone (*n* = 3). Table 3; Figure 2F present the baseline characteristics of these patients.

**TABLE 3:** Baseline status of patients receiving targeted treatment with disitamab vedotin.

	Gender	Age	HER2	Primary site	TNM stage	Metastasis
1	Male	51	2+	Bladder	T3N1M1b	Liver
2	Male	70	2+	Renal pelvis	T3N2M1	Bone
3	Female	76	1+	Bladder	T4aN1M0	None
4	Male	55	2+	Renal pelvis	T3N1N1	Lung, Bone
5	Male	63	2+	Bladder	T4bN0M0	None
6	Female	65	2+	Renal pelvis	T3N2M1	Supraclavicular lymph Node
7	Male	81	1+	Bladder	T4aN0M1	Lung
8	Female	65	1+	Bladder, Ureter	T2bN0M1	Inguinal lymph node
9	Female	66	2+	Ureter	TxN2M1	Abdomen, Liver
10	Female	66	2+	Renal pelvis	T3N2M1	Bone
11	Male	58	2+	Ureter	T3N2M0	None
12	Female	72	1+	Ureter	T3N0M1	Abdomen
13	Female	66	1+	Ureter	T3N1M1	Cervical lymph node
14	Female	56	1+	Renal pelvis	T2bN0M1	Liver
15	Male	66	2+	Bladder	T4aN3M1a	Distant lymph node
16	Male	78	2+	Bladder	T2bN0M0	None

**FIGURE 4:**
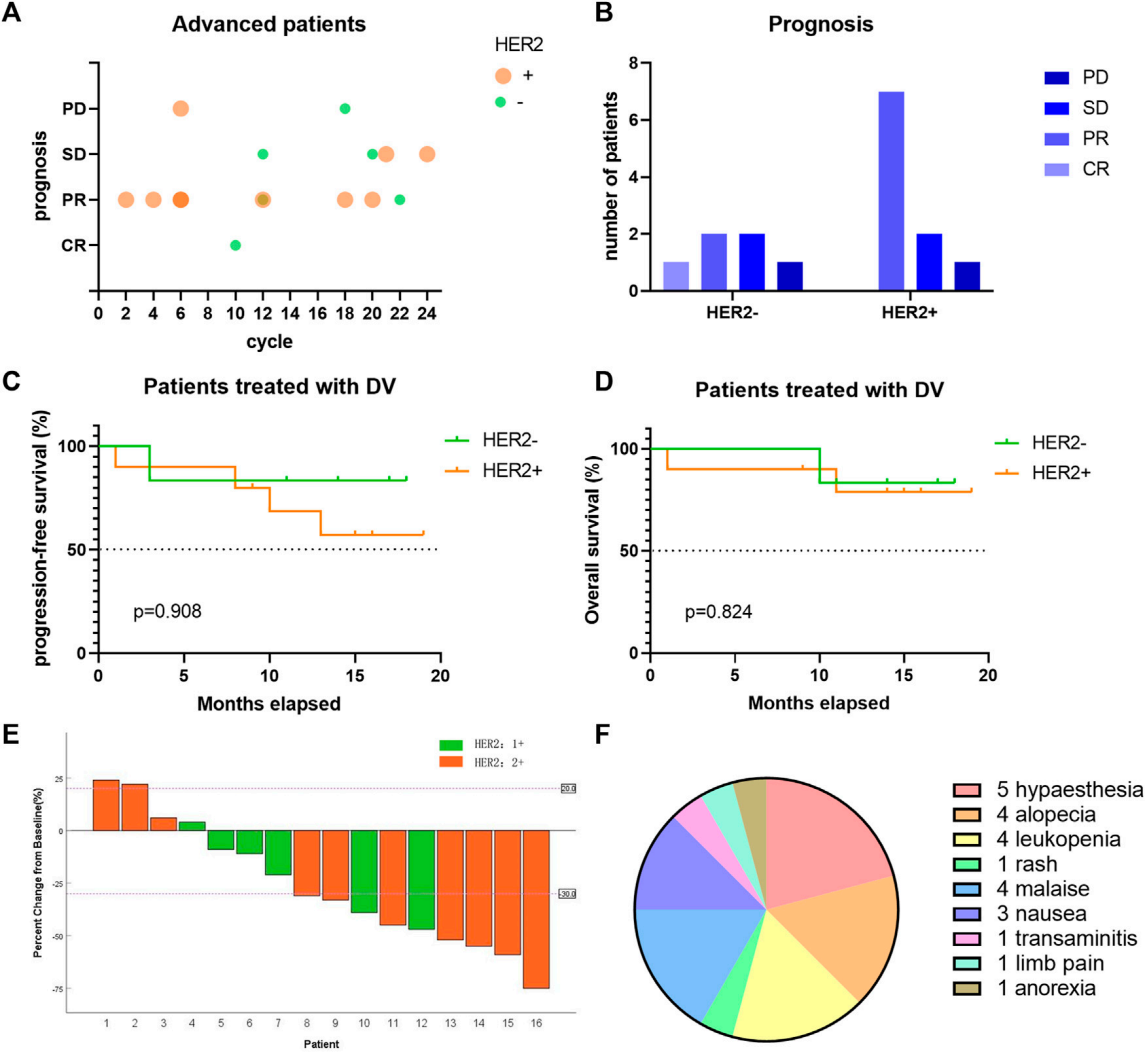
The efficacy of DV on the treatment of locally-advanced/metastatic UC patients. **(A)** Each bubble represents one patient, with different treatment cycle, HER2 expression and different prognosis. Bubbles with darker color reflected more than one patient. **(B)** The short-term prognosis of advanced UC patients divided by HER2 expression. **(C–D)** The long-term prognosis of advanced UC patients divided by HER2 expression. **(E)** Changes of patients’ tumor size compared to baseline. **(F)** Side effects of DV treatment in locally-advanced/metastatic UC. DV, disitamab vedotin; CR, complete response; PR, partial response; SD, stable disease; PD, progression disease.

After a median follow-up duration of 14 (1.0–19.0) months, we presented the treatment cycle and prognosis of each patient in Figure 4. According to RECIST 1.1 criteria, one patient achieved complete response (CR), while nine patients showed partial response (PR). Four patients exhibited stable disease (SD), and two patients experienced progressive disease (PD) as shown in Figures 4A, B. The ORR was found to be 62.5%, with tumor size reduction observed in twelve out of sixteen patients compared to baseline measurements, indicating a decrease rate of 75% as depicted in Figure 4E. The DCR was determined to be 87.5%. Among the ten HER2-positive patients expressing HER2 at level 2+, the ORR reached up to 70%. For six HER2-negative patients expressing HER2 at level 1+, the ORR was recorded as being at a rate of 50%. Median progression-free survival or overall survival has not been reached yet, as illustrated in Figures 4C, D.

### 3.5 Side effects

Eleven patients reported side effects following DV treatment, with the most frequently observed being hypaesthesia (5/16), alopecia (4/16), leukopenia (4/16), debilitation (4/16), and digestive tract symptoms such as nausea and anorexia (4/16) (Figure 4F). These adverse events were all classified as grade 1 or 2, with no occurrences of grade 3 or higher. Symptomatic treatment was administered to all patients.

## 4 Discussion

Urothelial carcinoma is the third most common cancer with HER2 overexpression, following breast and stomach cancer. Recent clinical trials have demonstrated the efficacy of HER2 targeted therapy in urothelial carcinoma. However, there is variability in the reported rates of HER2 positivity. A European study found that 4%–20% of urothelial carcinoma patients exhibited HER2 expression (Bellmunt et al., 2015). Cheetham and Petrylak (2016) reported a range of 5%–89% for bladder UCs overexpressing HER2 protein. In Chinese bladder UC patients, the expression of HER2 differs from other countries and also varies among provinces. Studies conducted in Beijing showed a HER2 positive rate ranging from 36.1% to 44% in UCs (Fan et al., 2022; Zhou et al., 2023). In our study conducted in Shandong Province, China, we observed that 159 out of 185 cases (86%) of high-grade UC expressed HER2 protein, with 127 cases exhibiting positive staining (IHC score 2+ and 3+), accounting for 68.6%. Furthermore, we found variations in HER2 expression among different primary sites within high-grade UCs; bladder UCs had a higher rate of positivity (74.8%) compared to ureters (55.6%) or renal pelvises (59.1%). The majority of cases exhibited moderate levels of HER2 expression at an IHC score of 2+ (110/185; 59%), while only a small percentage showed strong staining at an IHC score of 3+ (17/185; 9%). This was significantly lower than those with weak staining at an IHC score of 1+ (32/185; 17%) or moderate staining at an IHC score of 2+. Wide ranges of HER2+ reappearances have been observed in several studies, which can be attributed to suboptimal staining processes and the lack of standardized criteria specific for UC (Scherrer et al., 2022). The frequency of HER2 protein overexpression is influenced by multiple factors, including ERBB2 mutation and amplification. In muscle invasive bladder cancer, the expression rate of HERR2 amplified or single nucleotide variation (SNV) was found to be less than 20% (Kiss et al., 2017). Samples with ERBB2 amplification exhibited higher mRNA and protein expression levels. However, gene amplification alone does not solely drive high HER2 expression in bladder cancer; SNVs occur prior to ERBB2 amplification. SNVs occurring in the extracellular region of ERBB2 appear to result in lower protein expression detection. The practicality and high sensitivity of IHC-based HER2 detection still remain significant.

The detection of HER2 expression in urothelial carcinoma is not currently incorporated into routine clinical practice, thus the understanding of HER2 expression in urothelial carcinoma remains unclear. Despite the known overexpression of HER2 in various tumors, there are still conflicting data regarding its role as a carcinogenic driver or prognostic marker for urothelial carcinoma (Krüger et al., 2002; Bellmunt et al., 2015), Notably, treatment with ADC targeted HER2 has significantly improved survival rates for patients with HER2+ breast cancer and gastric cancer. Previous studies on the detection criteria for urothelial cancer HER2 mostly referred to breast cancer or gastric cancer, resulting in substantial variations among research findings. In 2021, China released the Expert Consensus on Clinical Pathology of Human Epidermal Growth Factor Receptor 2 Detection in Urinary Cancer which highlighted key differences between detection criteria for urothelial cancer and breast cancer. For urothelial cancer, scoring is defined as follows: 0 indicates non-staining or <10% incomplete and weak staining of infiltrating cancer cell membranes; 1+ denotes ≥10% incomplete and weak staining; 2+ signifies ≥10% weak to moderate staining of intact cell membrane or <10% strongly stained intact cell membrane; and finally, 3+ represents ≥10% strong staining of intact cell membranes.

A higher percentage of HER2+ expression is observed in high-grade UC. Bai et al. (2022) conducted immunohistochemical staining to evaluate HER2 expression in 108 patients with bladder UC who underwent radical cystectomy. They discovered that 57.4% of patients exhibited HER2 overexpression, which was significantly correlated with elevated tumor grade (*p* = 0.006) and staging (*p* < 0.001) (Bai et al., 2022). A meta-analysis by Zhao et al. (2015) also revealed a positive association between HER2 expression and high tumor grade. Similarly, Krüger et al. (2002) found that HER2 overexpression was more frequently detected in the high-grade cancer group compared to the low-grade cancer group among 138 bladder cancer cases. It should be noted that within the high-grade category, there are histological types associated with both better and poorer prognosis. Additionally, areas of high-grade UC specimens often exhibit negative tissue for HER2, indicating significant heterogeneity within UC, particularly in poorly differentiated tumors with a higher grade; thus suggesting that differences in HER2 overexpression may be linked to tumor heterogeneity.

According to our study, a significant difference was observed in the percentage of HER2+ among different clinical stages of UC (*p* = 0.019) (Table 2). This finding is consistent with another research study, which demonstrated a strong association between elevated levels of HER2 and the stage of UC at both mRNA and protein levels (*p* < 0.001) (Hussein et al., 2021). It is worth noting that HER2 overexpression is considered an early event in urothelial tumor development and rarely occurs during subsequent tumor progression. Therefore, there may be limited correlation with depth of myometrial invasion or lymph node metastasis (Goodman and Osunkoya, 2016). According to our current knowledge, there is limited literature discussing the association between blood indicators and HER2 expression in UC. However, relevant studies have been conducted in breast cancer. Specifically, red cell distribution width (RDW), RDW to platelet ratio (RPR), and platelet cell distribution width (PDW) have shown correlations with HER-2 expression in breast cancer tissues (Takeuchi et al., 2019).

Disitamab vedotin served as a second-line therapy for patients with locally-advanced or metastatic UC expressing HER2. In a phase II study involving 43 HER2-positive UC patients, the ORR was determined to be 51.2% after a follow-up period of 20.3 months, while the median PFS and OS were found to be 6.9 months and 13.9 months, respectively (Sheng et al., 2021a). Another clinical study reported an ORR of 46.9% and a median PFS of 4.3 months, with a median OS of 14.8 months (Sheng et al., 2021b). Apart from DV, there are several other HER2 ADCs that have also been applied in UC. Trastuzumab emtansine (TDM1), an ADC comprising the anti-HER2 antibody rastuzumab, has received FDA approval for treating HER2-positive bladder cancer patients who have previously undergone paclitaxel/rastuzumab therapy. In the phase II KAMELEON study (NCT02999672), patients with advanced urothelial bladder cancer positive for HER2 were included (de Vries et al., 2023). Following a median follow-up duration of 7.39 months (4.11–10.02) and a median exposure duration of 7.14 weeks for the metastatic UBC cohort, an overall response rate (90% CI) of 38.5% (16.57%–64.52%) to TDM1 was achieved. In total, 84.6% (11/13) of patients in the UC cohort experienced ≥1 adverse event, all considered treatment-related. Trastuzumab deruxtecan (DS-8201a) is another HER2 ADC compound composed of a spliceable linker connecting trastuzumab and an exatecan derivative acting as a topoisomerase I inhibitor. Notably, DS-8201a exhibits a higher drug-antibody ratio compared to TDM1, enabling its efficacy even in tumors with low HER2 expression. The ORR of DS-8201a was reported as 25% (4/16) in a phase I dose-escalation and dose-expansion study (Banerji et al., 2019). Considering the remarkable efficacy of disitamab vedotin, DV received approval from the National Medical Products Administration in January 2022. The extracellular domain of RC48 is a humanized anti-HER2 antibody that conjugates with microtubule protein inhibitors (MMAE) through a cleavable linker. MMAE released via enzymatic hydrolysis exhibits high membrane permeability and exerts therapeutic effects on tumor cells exhibiting low or no expression of HER2 (Padua et al., 2022). Accordingly, an ongoing follow-up study (NCT04073602) investigating RC48-ADC in patients with low HER2 expression enrolled a total of nineteen participants, revealing an ORR of 26.3%, median PFS of 5.5 months, and median OS of 16.4 months (Xu et al., 2022a). Our findings align closely with those observed in our study.

ICIs targeting PD-1 have demonstrated promising results in the treatment of bladder cancer, particularly in cases of metastatic UC that have progressed after chemotherapy. Recent studies have reported on the combination therapy of PD-1 and HER2 targeting ADCs. In a breast cancer model study, the combination of disitamab vedotin and PD-1 antibody exhibited remarkable efficacy in mice, surpassing the effects observed with either disitamab vedotin or PD-1 antibody alone. Furthermore, this combined treatment facilitated the formation of immunological memory, providing protection against tumor rechallenge (Huang et al., 2022). The observed infiltration of immune cells in mouse tumors following disitamab vedotin therapy suggests the potential for synergistic therapeutic effects by combining an immune checkpoint inhibitor (PD-1 inhibitor). Clinical data also supports this, with a report on the combination of RC48 and pembrolizumab in advanced UC (Xu et al., 2022b). The patient achieved CR and long-term PFS (>12 months). In a phase Ib/II study (RC48-C014), preliminary results demonstrated promising synergistic efficacy of RC48 in combination with toripalimab for advanced UC patients (Zhou et al., 2022a). The recommended dosage was RC48-ADC 2 mg/kg + toripalimab 3 mg/kg administered every 2 weeks. After a median follow-up of 8.0 months for 36 patients, the confirmed ORR was 76.7%. The median PFS at that time was 9.2 months, while the median OS had not been reached (Sheng et al., 2022). Another clinical study conducted in Fujian Province, China enrolled nine locally advanced or metastatic UC patients who were treated with DV combined with tislelizumab/toripalimab. After a median follow-up of 12 months, the ORR was found to be 88.9% (Wei et al., 2023). Additionally, ongoing clinical trials are investigating vedotin-tislelizumab as neoadjuvant treatment for HER2-positive locally advanced bladder urothelial carcinoma patients (Wen et al., 2024; Wen et al., 2022). In our study, the addition of DV was implemented following the failure of tislelizumab monotherapy. Notably, positive PD-L1 expression was observed in 20% of cases, while the efficacy of PD-1 alone exhibited limitations (Ma et al., 2023). Encouragingly, a clinical trial (RC48-C014) demonstrated that combining RC48-ADC with toripalimab yielded promising efficacy (ORR of 75% in all patients) for individuals with metastatic urothelial carcinoma (Zhou et al., 2022b), surpassing the outcomes achieved by DV monotherapy. Consequently, we opted for combination therapy involving disitamab vedotin and tislelizumab. In our study involving sixteen locally-advanced/metastatic UC patients, after a median follow-up of 14 months, the ORR among HER2+ patients was observed to be 70%, whereas it was found to be 50% among HER2-patients. Overall, the ORR reached up to 62.5% across all sixteen patients studied. These findings highlight the efficacy of RC48 not only in HER2+ but also in HER2- UC patients.

The RC48 exhibited manageable adverse effects in patients with UC. In our study, 68.8% (11/16) of the patients reported side effects, all of which were classified as grade 1 or 2. The most frequently reported side effects included hypaesthesia, hair loss, and leukopenia. In other studies on UC, patients experienced grade 1 or 2 side effects such as loss of appetite, rash, and fatigue (Wei et al., 2023), as well as grade 3 side effects including hypoesthesia and neutropenia (Sheng et al., 2021a). Other reported grade 3 side effects comprised anemia, hypoalbuminemia, urinary tract infection, and autoimmune encephalitis (Chen et al., 2023). All these side effects were effectively managed through appropriate treatments.

There are several limitations in our study. Firstly, the duration of follow-up was insufficient to obtain data on PFS or OS. In this paper, we solely investigated HER2 expression in UC based on the 2021 edition of the clinical pathological expert consensus on HER2 testing in UC in China. The association between HER2 positivity and long-term prognosis in UC patients remains unknown. Secondly, advanced patients received a combination therapy of DV and PD-1 inhibitors rather than DV alone, as our aim was to achieve improved patient outcomes. Further research is warranted to gain a better understanding of the long-term efficacy of DV monotherapy in UC patients. Although preclinical studies have shown promising results with RC48 used alongside PD-1/L1 inhibitors, additional evidence is required from clinical practice.

## 5 Conclusion

HER2 is a promising therapeutic target for UC, and its expression level holds critical significance in treatment response. Currently, HER2-targeting ADCs have demonstrated remarkable efficacy in select clinical trials (Zhou et al., 2023). However, the existing evaluation criteria for HER2 are inadequate for UC, leading to substantial discrepancies among research findings. Therefore, the establishment of a standardized scoring system is imperative to accurately identify individuals suitable for anti-HER2 ADC therapy and holds significant clinical implications. In this study, we adopted a novel Chinese standard to assess the expression rate of HER2 in high-grade UC patients with the aim of promoting uniformity in evaluating HER2 expression across UC studies. Our results indicate widespread protein expression of HER2 in urothelial carcinoma and reveal its close association with advanced stages of high-grade urothelial carcinoma. Targeting HER2 presents a potential therapeutic pathway for tumor management in UC patients. Combination therapy involving DV inhibitors and PD-1 blockade demonstrates both efficacy and acceptable side effects when treating advanced UC patients with either positive or negative HER2 status.

## Data Availability

The original contributions presented in the study are included in the article/Supplementary material, further inquiries can be directed to the corresponding author.
